# Breastfeeding related knowledge, attitudes, perceptions and practices of primary healthcare professionals in Ireland: A national cross-sectional survey

**DOI:** 10.1371/journal.pone.0320763

**Published:** 2025-04-09

**Authors:** Denise McGuinness, Kate Frazer, Sarah Brennan, Nancy Bhardwaj, Paula Cornally, Siobhan Ni Mhurchu, Marie Cantwell, Anne Pardy, Laura McHugh, Walter Cullen, Niamh Vickers

**Affiliations:** 1 School of Nursing, Midwifery and Health Systems, University College Dublin, Dublin, Ireland; 2 School of Medicine, University of Galway, Galway, Ireland; 3 School of Public Health, University College Dublin, Dublin, Ireland; 4 Health Service Executive, Ireland; 5 School of Medicine University College Dublin, Dublin, Ireland; Ateneo de Manila University Ateneo School of Medicine and Public Health, PHILIPPINES

## Abstract

**Background:**

Global research identifies the importance of breastfeeding, including the World Health Organisation in developing recommendations and noting over 800,000 child lives would be saved each year if breastfeeding was adopted following the recommendations of WHO/UNICEF. There is limited published data exploring breastfeeding knowledge, attitudes, perceptions and practices [KAPP] of health care professionals employed in primary care. Recent Irish evidence from one local geographical area identified general practitioners and general practice nurses [GPs and GPNs] received limited formal breastfeeding education within undergraduate or postgraduate education programmes and were interested in undertaking further professional development, education and training.

**Methods:**

Following ethical approval, a national cross sectional online survey using a breastfeeding [KAPP] survey instrument was completed using the Qualtrics platform. All registered GPs, GP trainees and General Practice Nurses [GPNs] in the Republic of Ireland were invited to participate. The online survey link was distributed via Ireland’s Health Service Executive health link email register via two senior HSE gatekeepers. Data collection was from June 1^st^ 2023, to November 17^th^, 2023.

**Results:**

A total of 662 primary health professionals participated, including 58.2% GPs, 14.2% GP trainees and 27.6% GPNs. The response rate to the survey was 10%, with approximately 6618 healthcare professionals receiving the link to the survey and 662 participating. Approximately 78% of respondents reported always recommending breastfeeding to women, and the majority (94.2%) were interested in completing further breastfeeding education. Barriers to training noted were time (84.3%), workload (62%) and financial cost (34.9%). Perceived and factual breastfeeding knowledge, perceived attitude and confidence scores with breastfeeding related issues significantly differed among the three groups.

**Conclusion:**

This national study reports low engagement with a national KAPP survey. There is inadequate preparation of primary healthcare professionals both theoretically and clinically to promote, protect and support breastfeeding in the primary healthcare setting, and has important implications for supporting wellbeing and shaping population health and achieving sustainable development goals.

## Introduction

Breastfeeding and the provision of human milk are important for short and long-term infant and maternal population health. The World Health Organisation (WHO) recommends that all infants are exclusively breastfed for the first six months of life and that breastfeeding is continued with the introduction of complementary foods until two years or beyond [1]. Research identifies the importance of breastfeeding, the risks of not breastfeeding [2] and it is a target of the Sustainable Development Goals [3]. It is suggested that almost 800,000 child lives would be saved each year following the WHO recommendations. Breastmilk provides macro and micronutrients including oligosaccharides, immunoglobulins, cytokines, lactoferrin, leucocytes and growth factors that are exclusive to human milk and cannot be replicated [4]. Infant benefits include less overweight and obesity, asthma and allergy [2], type 2 diabetes [5] and better cognition/IQ [6]. Exclusive breastfeeding for 90 + days is associated with reduced childhood morbidity [7].

Maternal benefits include the protective effect of breastfeeding against breast [8] and ovarian cancer, reducing the risk of type 2 diabetes, hypertension, cardiovascular disease [9,10] and osteoporosis [11]. Breastfeeding beyond one year (in addition to complementary foods), contributes significantly to improved health outcomes for both mother and child [12].

While we know that breastfeeding is recommended, the rates of breastfeeding vary globally. The Global Breastfeeding Scorecard (GBS) was introduced by the United Nations International Children’s Emergency Fund (UNICEF) and WHO to examine current breastfeeding practices worldwide and document key performance indicators. The Global Breastfeeding Scorecard (2023:2) records survey results from 2016–2022 and reports 46% of infants were breastfed within an hour of birth against a target of 70% for 2030 [13]. Ireland is acknowledged as having lower rates of “any breastfeeding since birth” at 60.8% [14] in comparison to the USA, 83% [15] and Sweden, 93% [16] with impactful implications for infant and maternal health. In Ireland there were approximately 57,540 births in 2022 [17] and by the age of three months 31.1% of Irish mothers were breastfeeding exclusively [14,18,19]. Recently the implementation of the revised Baby Friendly Hospital Initiative (BFI) commenced across all maternity hospitals in Ireland (n = 19). Internationally the BFI supports the implementation of the ‘Ten Steps to Successful Breastfeeding’ as standard care in maternity facilities: protecting, promoting and supporting breastfeeding. Additionally, Ireland engages with the World Breastfeeding Trends Initiative (WBTi) which assesses and monitors key breastfeeding policies and programmes [19]. The First WBTi report in Ireland was completed in 2023 by a group of academics, healthcare professionals and representatives of breastfeeding support groups. This WBTi report will provide a benchmark against future assessments and progress.

The majority of care and support for breastfeeding occurs in primary care in Ireland and it is acknowledged that better support is required for mothers [20–23]. While there are increased dedicated lactation posts in the Irish Health Service Executive (HSE) since 2023, more are required. Mothers living in more socially challenged areas and mothers of preterm and sick children must be supported by HCPs with breastfeeding knowledge and expertise. McGuinness *et al*. [24] report, from a feasibility study within one Irish urban community healthcare organisation, that 42.7% (n = 47/110) of general practitioners (GPs) and general practice nurses (GPNs) never attended a breastfeeding education programme and 53.9% (n = 55/102) identified that their knowledge could be improved. This represents structural barriers for those wishing to breastfeed [24]. The primary health care team, notably the GP and GPN, has a significant role to play, and little is known about their knowledge base and requirements to support breastfeeding. This paper reports from the first national survey of breastfeeding knowledge, attitudes, perceptions and practices (KAPP) of healthcare professionals employed in primary care in Ireland.

## Methodology

### Aim

The aim of this study was to describe and compare the breastfeeding knowledge, attitudes, perceptions and practices of GPs, GP trainees and GPNs employed in primary care in Ireland.

### Study design

An online anonymous cross-sectional survey of GPs, GP trainees and GPNs working within primary care in Ireland was completed. This was a collaboration between University College Dublin, Irish College of General Practitioners [ICGP] and the Irish Health Services.

### Survey instrument

A validated KAPP survey instrument developed by Theodoridis *et al*. [25] and was used with permission. The survey instrument was modified for the Irish setting in relation to syntax, and a section seeking information on breastfeeding skills was added pertinent to the primary health care professionals and comprised four sections. Section One reported demographic and practice characteristics, with no identifying information sought. Section Two included 15 statements to explore knowledge about breastfeeding and utilised a five-point Likert scale ranging from 1 (strongly disagree) to 5 (strongly agree). Section Three included 16 statements to explore attitudes and perceptions towards breastfeeding and utilised a five-point Likert scale also from 1 (strongly disagree) to 5 (strongly agree).

Section Four included four questions to explore confidence with clinical skills in relation to latch, breast and nipple challenges, supplementation, supporting lactation following preterm birth, and suppression of lactation following informed choice or neonatal death (Section four utilised a 3-point Likert scale: 1(not confident), 2 (somewhat confident), 3 (confident)). A final question was added to establish subsequent education and training requirements and the preferred mode of delivery, blended or in person.

### Inclusion criteria

All HSE registered General Medical Service (GMS) GPs, GP trainees and GPNs, were invited to participate in the study via the Irish Health System HSE Healthmail. All healthcare practitioners were qualified practitioners and registered to practice in the Republic of Ireland. The GP trainees were qualified medical doctors and pursuing specialist training in the post graduate GP trainee programme [26].

Participants may also have become aware of the research study via a GP or GPN or GP trainee colleague and/or social media. As this was a census survey, all GPs (4000 approx.) (House of the Oireachtas, 2022) [26] GPNs (2318) (NMBI 2023) [27] and GP Trainees (300 approx.) (House of the Oireachtas, 2022) [26] were invited to participate. In total approximately 6618 healthcare professionals received a link for the survey. Each professional category response rate is included in the results section.

### Data collection

The Strengthening the Reporting of Observational Studies (STROBE) guidelines [28] were used in the design and reporting of this study (S1 Appendix). The anonymous online survey was developed and distributed via the Qualtrics platform system. A participant information leaflet and written consent were embedded in the online survey, enabling informed consent, and this was completed before advancing to the full survey. A link to the survey was distributed via the HSE Health link email register via HSE gatekeepers initially who sent subsequent emails at regular intervals to strengthen knowledge and awareness of the research study. Recruitment was supported by the ICGP, the Irish General Practice Nurses Association and use of social media platforms. The survey link was also shared on a WhatsApp working groups of GPs, GPNs and GP Trainees. The data collection period ran from June 1^st^ 2023 to November 17^th^ 2023.

### Ethical approval

Ethics approval was granted in April 2023 from the ICGP Research Ethics Committee – Application no.: ICGP_REC_2023_010. An amendment to ethics was applied for and granted to increase the response rate using a work-related WhatsApp group text messaging service.

### Analysis

**Fig 1:**
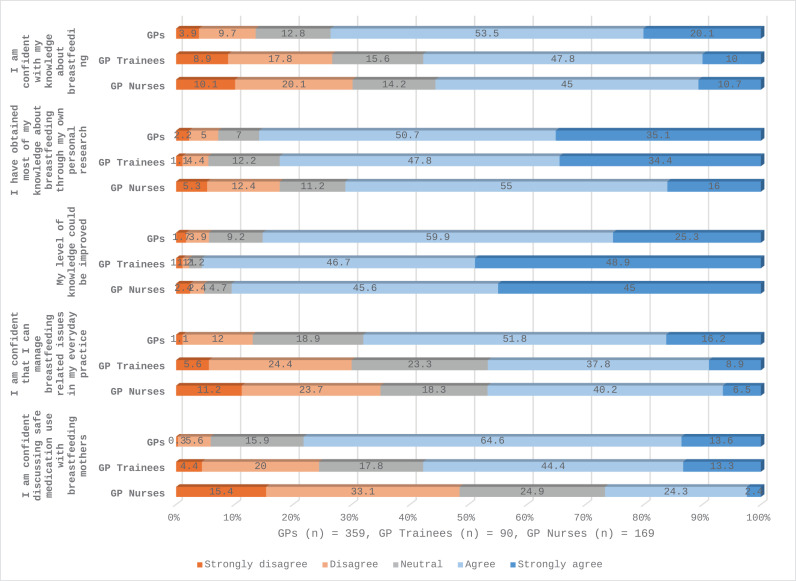
Perceived breastfeeding knowledge among GPs, GP trainees and GP Nurses.

**Fig 2:**
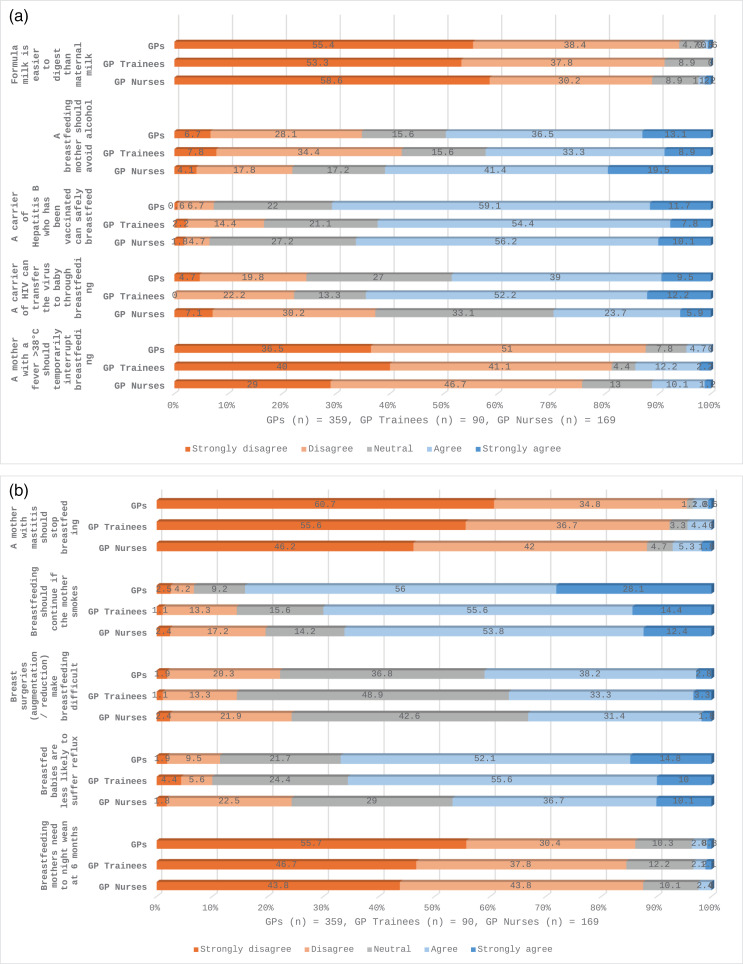
(a) and (b): Factual breastfeeding knowledge among GPs, GP Trainees and GP Nurses.

**Fig 3:**
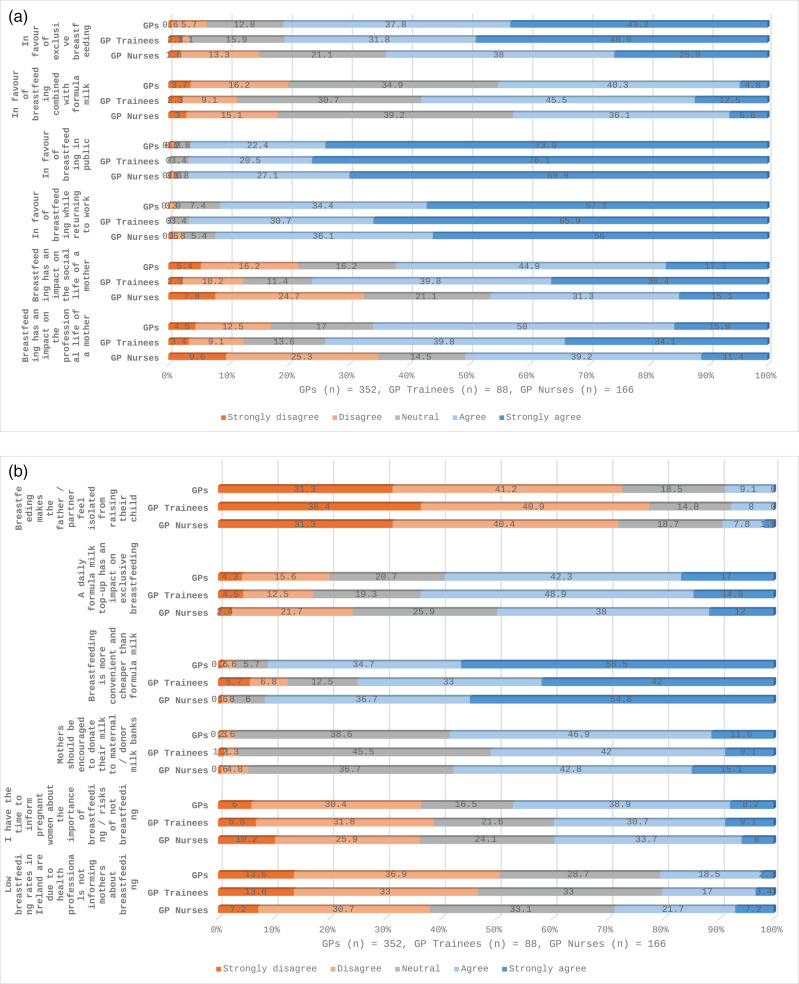
(a) and (b): Perceived attitudes and beliefs towards breastfeeding among GPs, GP trainees and GP Nurses.

**Fig 4:**
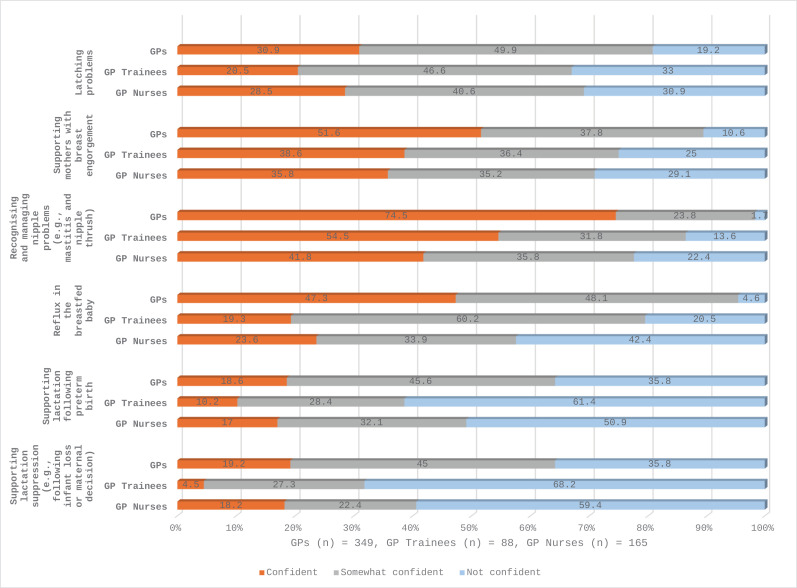
Perceived confidence for addressing breastfeeding related issues among GPs, GP trainees and GP nurses.

Statistical analysis of the anonymised data was performed using Statistical Package for Social Sciences (SPSS) version 27. Descriptive statistics were used to describe the participants using within group percentages and medians for ages and durations presented as text and table (Table 1). Breastfeeding related practices were described using group percentages (Table 2). Survey questions asked on Likert scale (covered questions on perceived and factual knowledge, breastfeeding perceptions/attitudes and perceived confidence with breastfeeding related issues) were analysed as percentages and response distribution to each Likert scale item within each group were presented as stacked bar graphs (Figs 1–4).

**Table 1:** Characteristics of Respondents.

Respondent characteristics	Overall % (n)	GPs % (n)	GP Trainees % (n)	GPNs % (n)
**Demographic characteristics (n = 662)**				
**Professional role**	100% (662)	58.2% (385)	14.2% (94)	27.6% (183)
**Age group n %**				
<40 years	45% (298)	41.6% (160)	93.6% (88)	27.3% (50)
41-60 years	48.2% (319)	50.6% (195)	6.4% (6)	64.5% (118)
>60 years	6.8% (45)	7.8% (30)	0% (0)	8.2% (15)
**Sex**				
Female	87.9% (582)	82.9% (319)	85.1% (80)	100% (183)
Male	12.1% (80)	17.1% (66)	14.9% (14)	0% (0)
**Education** ^*^				
Certificate	4.5% (30)	0.5% (2)	0% (0)	15.3% (28)
Degree	62% (410)	66.5% (256)	69.1% (65)	48.6^ (89)
Masters/Doctorate/Other	33.5% (222)	33% (127)	30.9% (29)	36.1% (66)
**Years in current role**				
Median (range)	5 (52)	7 (52)	1.5 (7)	5 (30)
Mean (SD)	8.55 (± 8.8)	10.37 (± 9.7)	1.97 (± 1.2)	8.09 (± 7.5)
**Years since first registered/graduated**				
Median (range)	16 (44)	17 (44)	6 (20)	23 (40)
Mean (SD)	18.5 (± 10.4)	19.03 (± 9.6)	7.12 (± 3.5)	23.25 (± 10.2)
**Completed breastfeeding education**	44.3% (293)	41.3% (159)	31.9% (30)	56.8% (104)
**Type of breastfeeding education completed (n = 293)**				
**As part of your undergraduate degree (medicine or nursing)/nurse education training programme**	39.2% (115)	44% (70)	36.7% (11)	32.7% (34)
**As part of post graduation education programme**	38.6% (113)	40.3% (64)	46.7% (14)	33.7% (35)
**Breastfeeding practical hands-on skills training**	18.1% (53)	11.3% (18)	10% (3)	30.8% (32)
**Continuous professional development programme**	32.1% (94)	28.3% (45)	23.3% (7)	40.4% (42)
**WHO/UNICEF (2020) Baby Friendly Hospital Initiative (BFHI) training course for maternity staff**	8.9% (26)	1.9% (3)	6.7% (2)	20.2% (21)
**HSELand Infant Feeding eLearning Modules**	19.5% (57)	10.7% (17)	6.7% (2)	36.5% (38)
**IBCLC**	3.1% (9)	2.5% (4)	3.3% (1)	3.8% (4)
**Completed either one or both of - breastfeeding practical hands-on skills training/ continuous professional development programme**	43.4% (126)	36.5% (58)	30% (9)	58.4% (59)
**Completed either one, two or all three of WHO/UNICEF BFHI Training course, HSELand Infant Feeding eLearning Modules, IBCLC**	25.9% (76)	13.8% (22)	13.3% (4)	48.1% (50)
**Further education on breastfeeding (n = 602)**				
**Interested in further education**	94.2% (567)	91.4% (319)	100% (88)	97% (160)
**Education on current recommendations (evidence-based)**	87.5% (496)	86.2% (275)	92% (81)	87.5% (140)
**Practical skill training to support mothers feed effectively**	73.5% (417)	70.5% (225)	83% (73)	74.4% (119)
**Method of training**				
**Online method of training**	52.4% (297)	55.8% (178)	43.2% (38)	50.6% (81)
**Face to face method of training**	17.6% (100)	13.8% (44)	22.7% (20)	22.5% (36)
**Blended method of training**	57.8% (328)	53.3% (170)	73.9% (65)	58.1% (93)
**Barriers to further breastfeeding training (n = 598)**				
**Time**	84.3% (504)	87.1% (305)	80.2% (69)	80.2% (130)
**Workload**	62% (371)	67.1% (235)	64% (55)	50% (81)
**Financial Cost**	34.9% (209)	30.3% (106)	31.4% (27)	46.9% (76)
**Other** ^**^	10% (60)	8.6% (30)	12.8% (11)	11.7% (19)

* Certificates were a traditional route for licensure for some nursing staff and some postgraduate certificate qualification is considered higher level than bachelors.

** Other barriers included: lack of support from employers; family life commitments, lack of options, travelling issues, mothers not being interested/give up early, near retirement, not confident about education provided in the past, do not perceive it as their domain/responsibility.

n represents number of responses to respective questions.

HSE, health service executive; IBCLC, international board certified lactation consultant

**Table 2:** Practices to support breastfeeding by GPs, GP trainees and GP Nurses.

Practices to support breastfeeding	Overall % (n)	GPs % (n)	GP Trainees % (n)	GPNs % (n)
Resources used to support breastfeeding for patients (n = 662)				
**Mychild.ie for signposting to families**	44.0% (291)	39.2% (151)	46.8% (44)	52.5% (96)
**Mychild.ie for updating my professional knowledge**	16.6% (110)	11.9% (46)	18.1% (17)	25.7% (47)
**Breastfeeding Observation and Assessment Tool (BOAT)**	3.9% (26)	2.9% (11)	0.0% (0)	8.2% (15)
**National Infant Feeding Policy for Primary Care Teams**	5.6% (37)	5.2% (20)	2.1% (2)	8.2% (15)
**The WHO BFHI Ten Steps to Successful Breastfeeding infographic**	8.9% (59)	6.2% (24)	11.7% (11)	13.1% (24)
**Referral to HSE PHN IBCLC (Lactation Consultant)**	45.0% (298)	48.8% (188)	47.9% (45)	35.5% (65)
**Referral to HSE PHN Led support group**	40.6% (269)	43.4% (167)	33.0% (31)	38.8% (71)
**Referral to Maternity Hospital IBCLC**	28.1% (186)	31.4% (121)	31.9% (30)	19.1% (35)
**Referral to Private IBCLC**	34.3% (227)	41.0% (158)	38.3% (36)	18.0% (33)
**Signposting or Referral to Voluntary breastfeeding support**	52.3% (346)	57.1% (220)	48.9% (46)	43.7% (80)
**Breast milk sample facilities** ^*^	0.9% (6)	0.5% (2)	1.1% (1)	1.6% (3)
**International Code of Marketing**				
**Awareness of International Code of Marketing (n = 662)**	48.2% (319)	51.2% (197)	38.3% (36)	47.0% (86)
**Adherence to International Code of Marketing (n = 318)**				
Yes	68.9% (219)	68.4% (134)	58.3% (21)	74.4% (64)
No	1.9% (6)	2.6% (5)	0.0% (0)	1.2% (1)
Unsure	29.2% (93)	29.1% (57)	41.7% (15)	24.4% (21)
**Breastfeeding recommendation**				
**Respondent children breastfed or intending to do in the future (n = 617)**				
Yes	88.8% (548)	90.5% (324)	85.6% (77)	87.0% (147)
Maybe	7.8% (48)	7.0% (25)	6.7% (6)	10.1% (17)
No	3.4% (21)	2.5% (9)	7.8% (7)	3.0% (5)
**Respondent recommends breastfeeding to mothers (n = 616)**				
Always	78.2% (482)	81.6% (292)	72.2% (65)	74.4% (125)
Sometimes	20.3% (125)	17.9% (64)	26.7% (24)	22.0% (37)
Never	1.5% (9)	0.6% (2)	1.1% (1)	3.6% (6)

n represents number of responses to respective questions.

WHO BFHI, World Health Organisation baby-friendly hospital initiative. PHN, public health nurse; HSE, health service executive; IBCLC, international board certified lactation consultant.

* Breast milk sample facilities – microbiology culture and sensitivity testing in collaboration with a hospital laboratory.

In addition to the description of breastfeeding KAPP’s and confidence with breastfeeding related issues among the three groups, statistical analysis to compare the three groups was conducted for Likert scale items as these included domains which could be influenced by subjective perspectives. This approach enabled presentation of distribution of responses, and in addition identifying statistically significant differences between groups which may guide more targeted interventions in breastfeeding education and support.

Likert-scale scores were treated as continuous variables and the mean scores on each Likert scale item were compared between the three groups [29,30]. Visual inspection of the distribution curve and measures of central tendency (mean, median) and measures of dispersion (standard deviation, interquartile range) revealed that the data was not perfectly distributed but was not highly skewed and fell within an acceptable range for parametric testing (S5, S7, S9 Tables). To formally test the data for normality, the Shapiro-Wilk test and the Kolmogorov-Smirnov test were applied which suggested deviation from normal distribution (S6, S8, S10 Tables). Nonetheless, Likert scale may not always exhibit a perfectly normal distribution, however, if the sample size is large enough parametric tests may still be robust to moderate deviations from normality [29,30]. Given the sufficiently large sample size in our study was adequate to invoke the central limit theorem, parametric tests were robust and employed to compare mean scores on Likert scale items between the three groups.

ANOVA was used to test for overall differences in mean Likert-scale scores across the three groups (GPs, GP Nurses, and GP Trainees) before adjusting for confounders. This provided a preliminary understanding of group differences. Linear regression was performed to adjust for confounders and helped to account for potential biases and provides a more nuanced understanding of factors influencing knowledge, attitudes, perceptions and confidence (S1, S2, S3, S4 Tables).

A linear regression model was used with Likert-scale scores (treated as continuous) as the outcome variable. Dummy variable regression included dummy variables for the categorical exposure variable of the three professional groups: GPs, GP Nurses, GP trainees. The dependent variables were the mean Likert-scale scores for each of the Likert scale items analysed separately (representing perceived and factual knowledge, attitude/perceptions and confidence) while exposure variables included the three professional groups with dummy coding, and GPs as the reference group.

Confounders included years in current employment and years since registration, as continuous variables. Completion of any breastfeeding education, recommendation of breastfeeding to mothers and personal breastfeeding experience or intention to do so were included as binary variables, for example, yes/no. P values of < 0.05 were considered statistically significant.

## Results

### Characteristics of the study respondents

A total of 662 respondents participated in the survey including 58.2% GPs, 14.2% GP trainees and 27.6% GPNs (Table 1). This represents approximately 10% of healthcare professionals approached for the study and eligible to participate (662/6618). The data represent 24 of the 26 counties in the Republic of Ireland, but no responses were recorded for counties Longford and Leitrim. The highest number of respondents were geographically located in counties Dublin, Cork, and Galway.

Table 1 presents the sociodemographic characteristics of respondents. Overall, 87.9% (n = 582) of respondents were female. The gender response, however, does not reflect the national distribution of GPs in Ireland. The majority of the GPs and GPNs were aged between 41 and 60 years; overall 48.2% (n =  319), with GPs 50.6% (n =  195) and GPNs 64.5% (n = 118). We report that 45% of respondents were under 40 years, with the majority of GP trainees (93.6%, n =  88) falling into this category. The median duration working in a clinical practice role was 5 years (mean 8.55 years) and the median time since first registration was 16 years (mean 18.5 years).

### Breastfeeding knowledge

In relation to breastfeeding knowledge, overall, 44.3% (n =  293) of respondents had completed a breastfeeding education programme; with 56.8% GPNs completing breastfeeding education compared to 41.3% (n = 159) GPs and 31.9% (n =  30) GP trainees, as shown in Table 1.

Overall, only 18.1% (n =  53) of respondents reported receiving any hands-on training in breastfeeding skills. Similarly, a sizable majority of 91.1% (n = 267) reported that they had never completed the WHO/UNICEF (2020) Baby Friendly Hospital Initiative Training Course for Maternity Staff and 80.5% (n =  236) never completed the online asynchronous Irish e-learning programme “HSELand Infant feeding e- Learning modules” provided nationally.

Our findings reported in Table 1 show that the majority of respondents (94.2%, n =  567) were interested in completing additional learning on breastfeeding; with 73.5% (n =  417) interested in learning more about practical breastfeeding skills and 57.8% preferred learning via a blended mode of education. Barriers to engagement in education programmes identified by respondents included time (84.3%, n =  504), workload (62%, n =  371) and the financial cost (34.9%, n =  209).

### Practices to support breastfeeding

Overall, the most common resources used by respondents to support breastfeeding among mothers included signposting to the HSE website online resource “MyChild.i.e.,” (44%, n =  291) (MyChild.i.e., is an Irish health service HSE online pregnancy and child health information site for parents), and referrals to: 1) HSE Public Health Nurse who is a registered lactation consultant (45%, n =  298), 2) HSE Public Health Nurse Led support group (40.6%, n =  269), 3), Maternity Hospital Midwife specialist/ International Board Certified Lactation Consultant (IBCLC) (28.1%, n =  186) and 4, or private IBCLC (34.3%, n =  227), shown in Table 2.

Approximately 48% (n =  319) identified their awareness of the International Code of Marketing of Breastmilk Substitutes (Table 2). Of this group 68.9% (n =  219) reported adherence to the International Code of Marketing of Breastmilk Substitutes, and approximately 30% (n =  99) were unsure if they did adhere to the Code. Overall, 88.8% (n =  548) of respondents identified that they had breastfed/or intended to breastfeed their own children and 78.2% (n =  482) reported always recommending breastfeeding to mothers.

### Perceived breastfeeding knowledge among GPs, GP Trainees and GPNs

Fig 1 and Table S1 present perceived breastfeeding knowledge among GPs, GP trainees and GPNs. Table S1 shows that GPs report significantly higher confidence in their knowledge of breastfeeding (Mean 3.76, SD 1) when compared to GP trainees (Mean 3.32, SD 1.15) and GPNs (Mean 3.26, SD 1.19). Significantly GPs report higher confidence in managing breastfeeding-related issues (Mean 3.7, SD 0.9) compared to GP trainees (Mean 3.2, SD 1.08) and GPNs (Mean 3.07, SD1.16) (p <  0.01). Regression analyses confirms a statistically significantly lower confidence score in breastfeeding knowledge among GP trainees (β =  -0.302, p <  0.05) and GPNs (β =  -0.669, p <  0.01) compared to GPs; and a lower confidence in managing breastfeeding-related issues among GP trainees (β =  -0.341, p <  0.01) and GPNs (β =  -0.761, p <  0.01) when compared to GPs.

All groups perceived a need to improve their breastfeeding knowledge, with GP trainees reporting the highest perceived knowledge needs at 48.9%, (n = 44) (Fig 1) (β =  0.276, p <  0.01) (Table S1)., while 25.2% (n = 90) of GPs strongly agreed with this statement. We report that 35.1% (n = 125) of GPs and 34.4% (n = 30) of GP trainees strongly agreed that they had obtained most of their knowledge about breastfeeding through their own personal research, with GPN results reporting this finding at 16% (n = 27) (Fig 1).

### Factual breastfeeding knowledge among GPs, GP trainees and GPNs

Significant differences in factual knowledge of breastfeeding were reported for respondents (Fig 2 (a), 2 (b) and Table S2). GPs achieved higher knowledge scores for five of the ten questions. Breastfeeding knowledge scores were significantly low for all respondents in relation to management of mastitis (Table S2). GPs’ factual knowledge (Table S2) was significantly higher for nine of the ten knowledge statements compared to the other groups:avoid alcohol when breastfeeding (β =  3.542, p <  0.01), hepatitis B and safe breastfeeding (β =  2.944, p <  0.01), HIV transmission through breastfeeding (β =  4.009, p <  0.01), interrupting breastfeeding due to a high temperature (β =  3.004, p <  0.01), breastfeeding with mastitis (β =  2.409, p <  0.01), breastfeeding and smoking (β =  3.208, p <  0.01), breast surgeries impact on breastfeeding (β =  2.934, p <  0.01), reflux in breastfed babies (β =  2.705, p <  0.01) and night weaning at 6 months (β =  1.911, p <  0.01). We report that 52% (n = 187) of GPs and 55.6% (n = 50) of GP trainees agreed that breastfed babies are less likely to suffer reflux (Fig 2 (b)). All respondents stated that they strongly disagreed/disagreed that formula milk was easier to digest than maternal milk, with results as follows: GPs (93.8%, n = 337), GP trainees (91.1%, n = 81) and GPNs (88.8%, n = 150) (Fig 2(a)). Similar results across all three professional groups are reported when asked if a breastfeeding mother should night wean at six months (Fig 2 (b)).

### Perceived attitudes and beliefs about breastfeeding among GPs, GP trainees and GPNs

Varying attitudes and beliefs about breastfeeding are reported by respondents (Fig 3 (a), 3 (b) and Table S3) GPs and GP trainees showed strong support for exclusive breastfeeding, with mean scores of 4.17 (SD 0.9) and 4.24 (SD 0.92), respectively, compared to GPNs (Mean 3.73, SD 1.04) (p <  0.01). Regression analyses confirmed higher attitudinal scores among GPs (β =  2.822, p <  0.01). GPs (Mean 3.2, SD 0.9) and GPNs (Mean 3.2, SD 0.9) were less in favour of breastfeeding combined with commercial formula milk when compared to GP trainees (Mean 3.57, SD 0.9) (p =  0.018). General Practice Nurses perceived a lesser impact of breastfeeding on mothers’ social and professional lives than GPs and GP trainees (Fig 3a) (Table S3). GPs (Mean 4.44, SD 0.764) and GPNs (Mean 4.43, SD 0.742)) perceived breastfeeding to be more convenient and less expensive than using formula milk compared to GP trainees (Mean 3.99, SD1.16) (p < 0.01). Support for breastfeeding in public and for breastfeeding while returning to work was high for all respondents with no significant differences reported between groups (Fig 3(a)) (Table S3). Similarly, all respondents disagreed with the statement that they have time to inform pregnant women about the importance of breastfeeding and the risks of not breastfeeding (Fig 3(b)).

### Perceived confidence with breastfeeding related issues among GPs, GP trainees and GPNs

There were significant differences on perceived confidence for all statements in this section of the survey instrument (Fig 4 and Table S4). Overall, GPs had higher confidence scores compared to the other groups. Standard deviation and mean confidence scores were lower for all respondents in supporting lactation following a preterm birth and for supporting lactation suppression following infant loss or maternal decision to stop breastfeeding. Similarly, lower confidence was reported supporting breastfeeding mothers with latching problems, with results reported as follows: GPs (30.9%, n = 108), GP trainees (20.5%, n = 18) and GPNs (28.5%, n = 48) (Fig 4).

## Discussion

The national study aimed to establish breastfeeding KAPP’s of GPs, GPNs and GP trainees in the Republic of Ireland. We identified that breastfeeding education provided to this cohort of primary healthcare professionals was suboptimal with significant differences reported in breastfeeding related knowledge and beliefs. The majority of respondents supported breastfeeding, promoted breastfeeding and were motivated to complete additional education and training. We acknowledge that the response rate nationally was very low despite the support of theIrish Health Service Executive and professional bodies and while the gaps in knowledge exist for those who did reply we are unsure if it is the same for those who did not participate. Breastfeeding education and training are mandatory for undergraduate, postgraduate Midwifery students and Public Health Nursing students in Ireland. However, it is evident from the results of this study that GPs and GPNs are not provided with standard breastfeeding education and skills and importantly the information to manage different breastfeeding challenges within their respective education programmes. The WBTi (2023) Assessment Report in Ireland recommends the provision of independent evidenced based breastfeeding education, training and competency assessment for all healthcare professionals that engage with the breastfeeding dyad [19] and the gaps in knowledge noted in this study would support the recommendation. In our study, the respondents also reported reduced confidence in supporting mothers in suppressing lactation following infant loss.

Key time frames and support providing education to sustain breastfeeding includes the period following discharge from the hospital [31]. The GP, GP trainee and GPN, as members of the primary health care team, are in a prime position to improve health outcomes for women and infants by promoting and protecting breastfeeding, having the confidence, knowledge and skills to support breastfeeding parents and through understanding of available community resources locally. They are the key to changing societal influences on breastfeeding [32].

It is estimated that a GP will engage with a healthy pregnant woman at least six times during pregnancy and with two additional post-natal consultations, as part of the Irish HSE Maternity and Infant Care Scheme [33]. Primary healthcare professionals must be knowledgeable to support lactation for the breastfeeding dyad to meet SDG goals [3,25,33,34]. A lack of breastfeeding and lactation training among community healthcare professionals exposes mothers to receiving inconsistent breastfeeding advice, which can contribute to early weaning practices [35,36]. Our research identified that while GPs had higher self-reported and actual breastfeeding knowledge and higher confidence scores, reported attitudes towards breastfeeding varied among respondents. Interestingly, many of the respondents noted using personal experience to support mothers, consistent with previous research [24,35,37,38].

Globally, the BFI states the importance of breastfeeding knowledge and skills for healthcare professionals, and this is recognised as a positive intervention to increase breastfeeding and exclusive breastfeeding rates [39–41]. An interdisciplinary team approach is necessary to both implement the BFHI, in addition to improved post discharge care to increase the duration of breastfeeding [40,41]. The Baby Friendly Community Initiative (BFCI) is an extension of the BFI and supports breastfeeding education and training in primary care [19,20]. Our study identified that the WHO BFI Ten Steps to Successful Breastfeeding was not a resource utilised to support breastfeeding families, which is a missed opportunity to support infant feeding intention and initiation discussions during the antenatal period. International evidence confirms compliance with the BFHI increased breastfeeding rates significantly among black women in the Southern United States [42], offering potential opportunities for Ireland to improve their breastfeeding rates. Ireland is engaging with the global BFI quality improvement programme which aligns with the HSE breastfeeding action plan, with no cost incurred to individual maternity units [19].

Breastfeeding and lactation knowledge deficits among primary healthcare professionals is not specific to Ireland. Mothers in Brazil are challenged with the care provided by their primary health care provider, reporting unsatisfactory breastfeeding support and incorrect or inadequate treatment of medical problems related to breastfeeding [43]. Interestingly, de Almeida *et al*. [44] integrative review of evidence notes healthcare professionals may consider breastfeeding as a natural and instinctive activity, and while many possess theoretical knowledge, they lack the practical skills necessary to support the breastfeeding dyad. Sandhi *et al*. [45] describe the importance of skills lab teaching and clinical practicum in breastfeeding education as a significant moderator to knowledge and skills. We identified that respondents in this study wanted to participate in further education and practical skills training. This is similar to Biggs *et al*. [33], which found that UK medical schools are not adequately preparing students to support breastfeeding families. Integrating lactation education within nursing, midwifery and medical programmes is essential to improving knowledge, attitude and skills [37,45,46]. In Lebanon, Moukarzel *et al*. [38], suggest undergraduate medical students experience a medical training programme with a focus on pathophysiology and medical treatment and with less focus on disease prevention and health promotion. They also highlight that many students relied on informal personal networks to learn about breastfeeding practices [38]. Moukarzel *et al.* [38], also states ethical concerns in relation to commercial milk formula companies offering incentives to paediatricians and obstetricians/gynaecologists to promote products. It is important that all healthcare professionals are aware of the impact of the commercial determinants of health and the International Code of Marketing of Breastmilk Substitutes to protect and promote appropriate infant and young child feeding practices [47]. The code provides a set of recommendations to regulate the aggressive and inappropriate marketing of breastmilk substitutes [47]. While approximately half of the GP and GPN participants in our study were aware of the International Code of Marketing of Breastmilk Substitutes, almost 30% (n = 93) were unsure if they adhered to the Code; approximately two thirds of GP trainees responded that they were not aware of the International Code of Marketing of Breastmilk Substitutes. Gaps in knowledge exist, and further education is necessary on the Code due to the implications for future maternal and child health outcomes.

The early breastfeeding weeks are not without challenges for the new breastfeeding dyad, especially with the introduction of artificial commercial milk. The introduction of formula milk during this critical period impacts exclusive breastfeeding, and the process of lactogenesis as a mother establishes a full milk supply; exclusive breastfeeding is associated with improved health outcomes [12,48]. Our study sought to identify attitudes and perceptions related to exclusive breastfeeding and if a daily formula milk top-up impacted exclusive breastfeeding. While GPs and GP trainees showed strong support for exclusive breastfeeding, GPs and GPNs were less in favour of breastfeeding combined with formula milk.

Professional knowledge and support for breastfeeding and lactation is important for all health care professionals, independent of gender. The majority of respondents in our study were female which is consistent with similar studies [33,49,50]. Notwithstanding the fact that healthcare has a female dominance, all the primary healthcare team, regardless of gender require the necessary theoretical and skills training to support the breastfeeding dyad.

Respondents in our study were knowledgeable about the importance of breastfeeding support groups. It is recognised that mothers benefit from joining support groups where breastfeeding experiences are shared in addition to professional support, particularly when the breastfeeding duration is challenged [44,51–53]. Primary health care professionals are in a privileged position to normalise breastfeeding, change societal attitudes, and impact climate change and sustainability [32]. Professional knowledge and skills are also required to initiate and sustain breastfeeding practices among women with low socioeconomic status and minority and immigrant women to reduce long-term health inequities in this population group, and consistent with achieving the SDGs [3,49,50,54–56]. Strategies are needed to support breastfeeding, particularly among women and families where there are challenges to initiate and sustain breastfeeding [57]. Quintero *et al*. [58] found breastfeeding disparities among diverse racial/ethnic groups despite public health efforts in the USA. Breastfeeding information from a doctor had limited or no effect on breastfeeding initiation or duration. It is recognised that women are influenced by their support networks, in addition to the support of a partner [52,59,60], but where this information is lacking breastfeeding women with challenges are at a disadvantage.

It is evident from the literature that breastfeeding support for many women ceased or availability was limited during the COVID 19 pandemic, and this may have impacted women’s future choices [61]. Fouladi *et al*. [62] notes the absence of patient and public engagement impacted public health at this time. Breastfeeding and lactation education programmes for healthcare professionals in the community upholds parents and children’s rights to meet their breastfeeding goals [32] with the assistance of knowledgeable and educated healthcare professionals. We hope that our national study will inform public policy and professional education programmes for GPs, GP trainees and GPNs to support their important role in normalising breastfeeding in primary care and improving health outcomes for all women and infants.

### Limitations

Ireland is a country with low breastfeeding rates. The response rate was low; approximately 6618 healthcare professionals received the link to the survey, and 662/6618 participated in the survey. This is despite the support of the HSE gatekeepers in engaging with HSE professional bodies and highlighting the importance of the study and the direct support from the Irish General Practice Nurse Association and Irish College of General Practitioners. We do not know why 90% of eligible healthcare professionals did not participate. It may be that healthcare professionals interested in breastfeeding participated in the survey. There was a limited response from practitioners who were not female across all included cohorts. We acknowledge that primary healthcare professionals are experiencing an increased workload which may account for the low response rate. The lack of response may also indicate the lack of education and training and the deprioritization of this subject in curricula. This was an anonymous online survey, and the response rate may not include those who could not participate at the time, and the data represent 24 of the 26 counties in Ireland. However, information and communication to participate were provided over a six-month period. We acknowledge that the results may under or overestimate breastfeeding knowledge, attitude, perceptions and skills and may not be generalizable to other countries and community primary healthcare practitioners.

## Conclusion

Results from this first national study in the Republic of Ireland suggest inadequate educational preparation in Irish medical and nursing schools and highlight deficiencies in supporting women with lactation and breastfeeding. Breastfeeding is a global public health measure and within the primary healthcare team, requires that healthcare professionals are provided with access to education and training to support women and infants, increase national breastfeeding rates and attain SDGs.

## Supporting information

S1 Appendix Strobe Guideline. (DOC)

S1 Table Perceived breastfeeding knowledge among GPs, GP trainees and GPNs. (DOCX)

S2 Table Factual breastfeeding knowledge among GPs, GP trainees and GPNs. (DOCX)

S3 Table Perceived attitudes and beliefs about breastfeeding among GPs, GP trainees and GPNs. (DOCX)

S4 Table Perceived confidence with breastfeeding related issues among GPs, GP trainees and GPNs. (DOCX)

Table S5-S10 Testing normal distribution for Likert scale items. (DOCX)
