# Supplementary material for: Breastfeeding related knowledge, attitudes, perceptions and practices of primary healthcare professionals in Ireland: A national cross-sectional survey
Source: PLoS One. 2025 Apr 9;20(4):e0320763. doi: 10.1371/journal.pone.0320763 (PMC11981121; doi:10.1371/journal.pone.0320763)
Supplement: S1 Table — (DOCX) [file pone.0320763.s002.docx]

**S 1 Table : Perceived breastfeeding knowledge among GPs, GP trainees and GP nurses**

| **Item/Variable** | **Professional role** | **N** | **One-way Anova** | | **Regression analysis**** | | | |
| --- | --- | --- | --- | --- | --- | --- | --- | --- |
|  |  |  | **Mean (SD)** | **p value** | **Unstandardized β Coefficients** | **Std. Error** | **t** | **p value** |
| **a) I am confident with my knowledge about breastfeeding** | GP | 359 | 3.76 (1.008) | <0.01* | 2.148 | 0.407 | 5.283 | <0.01 |
|  | GP Trainee | 90 | 3.32 (1.15) |  | -0.302 | 0.135 | -2.232 | 0.026 |
|  | GP Nurse | 169 | 3.26 (1.192) |  | -0.669 | 0.108 | -6.167 | <0.01 |
|  | Total | 618 | 3.56 (1.106) |  |  |  |  |  |
| **b) I have obtained most of my knowledge about breastfeeding through my own personal research** | GP | 359 | 4.11 (0.901) | <0.01* | 4.061 | 0.363 | 11.197 | <0.01 |
|  | GP Trainee | 90 | 4.1 (0.862) |  | -0.088 | 0.121 | -0.726 | 0.468 |
|  | GP Nurse | 169 | 3.64 (1.061) |  | -0.433 | 0.097 | -4.473 | <0.01 |
|  | Total | 618 | 3.98(0.964) |  |  |  |  |  |
| **c) My level of knowledge could be improved** | GP | 359 | 4.03 (0.808) | <0.01* | 4.579 | 0.313 | 14.648 | <0.01 |
|  | GP Trainee | 90 | 4.41 (0.701) |  | 0.276 | 0.104 | 2.647 | <0.01 |
|  | GP Nurse | 169 | 4.28 (0.853) |  | 0.319 | 0.083 | 3.825 | <0.01 |
|  | Total | 618 | 4.16 (0.819) |  |  |  |  |  |
| **d) I am confident that I can manage breastfeeding related issues in my everyday practice** | GP | 359 | 3.7 (0.918) | <0.01* | 2.336 | 0.384 | 6.082 | <0.01 |
|  | GP Trainee | 90 | 3.2 (1.083) |  | -0.341 | 0.128 | -2.668 | <0.01 |
|  | GP Nurse | 169 | 3.07 (1.163) |  | -0.761 | 0.102 | -7.428 | <0.01 |
|  | Total | 618 | 3.45 (1.054) |  |  |  |  |  |
| **l) I am confident discussing safe medication use with breastfeeding mothers** | GP | 359 | 3.86 (0.724) | <0.01* | 3.297 | 0.342 | 9.634 | <0.01 |
|  | GP Trainee | 90 | 3.42 (1.091) |  | -0.334 | 0.114 | -2.930 | <0.01 |
|  | GP Nurse | 169 | 2.65 (1.081) |  | -1.337 | 0.091 | -14.645 | <0.01 |
|  | Total | 618 | 3.46 (1.032) |  |  |  |  |  |
| *One-way ANOVA  **regression model adjusted for years in current employment and since registration, completed any breastfeeding education, recommend breastfeeding to mothers, breastfed own children or intend to do so in the future  p significant <0.05  all 5-point Likert scale items; higher mean score indicates higher perceived knowledge | | | | | | | | |
