# Supplementary material for: Breastfeeding related knowledge, attitudes, perceptions and practices of primary healthcare professionals in Ireland: A national cross-sectional survey
Source: PLoS One. 2025 Apr 9;20(4):e0320763. doi: 10.1371/journal.pone.0320763 (PMC11981121; doi:10.1371/journal.pone.0320763)
Supplement: S2 Table — (DOCX) [file pone.0320763.s003.docx]

**S 2 Table: Factual breastfeeding knowledge among GPs, GP trainees and GP nurses**

| **Item/Variable** | **Professional role** |  | **One-way Anova** | | **Regression analysis**** | | | |
| --- | --- | --- | --- | --- | --- | --- | --- | --- |
|  |  | **N** | **Mean (SD)** | **p value** | **Unstandardized β Coefficients** | **Std. Error** | **t** | **p value** |
| **e) Formula milk is easier to digest than maternal milk** | GP | 359 | 4.47 (0.68) | 0.845* | 4.005 | 0.276 | 14.489 | <0.01 |
|  | GP Trainee | 90 | 4.44 (0.655) |  | 0.023 | 0.092 | 0.254 | 0.800 |
|  | GP Nurse | 169 | 4.44 (0.8) |  | -0.069 | 0.074 | -0.935 | 0.350 |
|  | Total | 618 | 4.46 (0.711) |  |  |  |  |  |
| **f) A breastfeeding mother should avoid alcohol** | GP | 359 | 3.21 (1.182) | <0.01* | 3.542 | 0.440 | 8.042 | <0.01 |
|  | GP Trainee | 90 | 3.01 (1.166) |  | 0.116 | 0.147 | 0.789 | 0.430 |
|  | GP Nurse | 169 | 3.54 (1.118) |  | 0.235 | 0.117 | 1.999 | <0.05 |
|  | Total | 618 | 3.27 (1.174) |  |  |  |  |  |
| **g) A carrier of Hepatitis B who has been vaccinated can safely breastfeed** | GP | 359 | 3.75 (0.769) | 0.04* | 2.944 | 0.310 | 9.496 | <0.01 |
|  | GP Trainee | 90 | 3.51 (0.915) |  | -0.200 | 0.103 | -1.933 | 0.054 |
|  | GP Nurse | 169 | 3.68 (0.79) |  | -0.104 | 0.083 | -1.262 | 0.208 |
|  | Total | 618 | 3.69 (0.8) |  |  |  |  |  |
| **h) A carrier of HIV can transfer the virus to her baby through breastfeeding** | GP | 359 | 3.29 (1.038) | <0.01* | 4.009 | 0.399 | 10.040 | <0.01 |
|  | GP Trainee | 90 | 3.54 (0.973) |  | 0.153 | 0.133 | 1.153 | 0.249 |
|  | GP Nurse | 169 | 2.91 (1.028) |  | -0.362 | 0.107 | -3.402 | <0.01 |
|  | Total | 618 | 3.22 (1.046) |  |  |  |  |  |
| **i)A mother with a fever &gt; 38C should temporarily interrupt breastfeeding** | GP | 359 | 4.19 (0.773) | <0.01* | 3.004 | 0.329 | 9.134 | <0.01 |
|  | GP Trainee | 90 | 4.04 (1.07) |  | -0.255 | 0.109 | -2.327 | <0.05 |
|  | GP Nurse | 169 | 3.92 (0.964) |  | -0.274 | 0.088 | -3.125 | <0.01 |
|  | Total | 618 | 4.1 (0.882) |  |  |  |  |  |
| **j) A mother with mastitis should stop breastfeeding** | GP | 359 | 1.48 (0.712) | <0.01* | 2.409 | 0.296 | 8.145 | <0.01 |
|  | GP Trainee | 90 | 1.57 (0.765) |  | 0.174 | 0.098 | 1.768 | 0.078 |
|  | GP Nurse | 169 | 1.75 (0.907) |  | 0.208 | 0.079 | 2.638 | <0.01 |
|  | Total | 618 | 1.56 (0.785) |  |  |  |  |  |
| **k) Breastfeeding should continue if the mother smokes** | GP | 359 | 4.03 (0.876) | <0.01* | 3.208 | 0.354 | 9.054 | <0.01 |
|  | GP Trainee | 90 | 3.69 (0.92) |  | -0.252 | 0.118 | -2.135 | 0.033 |
|  | GP Nurse | 169 | 3.57 (0.992) |  | -0.443 | 0.095 | -4.684 | 0.000 |
|  | Total | 618 | 3.85 (0.938) |  |  |  |  |  |
| **m) Breast surgeries i.e., augmentation or reduction make breastfeeding difficult** | GP | 359 | 3.19 (0.859) | 2.42* | 2.934 | 0.328 | 8.954 | <0.01 |
|  | GP Trainee | 90 | 3.24 (0.769) |  | 0.116 | 0.109 | 1.063 | 0.288 |
|  | GP Nurse | 169 | 3.08 (0.834) |  | -0.174 | 0.087 | -1.995 | 0.046 |
|  | Total | 618 | 3.17 (0.84) |  |  |  |  |  |
| **n) Breastfed babies are less likely to suffer reflux** | GP | 359 | 3.68 (0.906) | <0.01* | 2.705 | 0.360 | 7.509 | <0.01 |
|  | GP Trainee | 90 | 3.61 (0.908) |  | -0.128 | 0.120 | -1.064 | 0.288 |
|  | GP Nurse | 169 | 3.31 (0.988) |  | -0.386 | 0.096 | -4.013 | <0.01 |
|  | Total | 618 | 3.57 (0.942) |  |  |  |  |  |
| **o) Breastfeeding mothers need to night wean at 6 months** | GP | 359 | 1.63 (0.842) | 0.381* | 1.911 | 0.316 | 6.046 | <0.01 |
|  | GP Trainee | 90 | 1.73 (0.845) |  | 0.125 | 0.105 | 1.188 | 0.235 |
|  | GP Nurse | 169 | 1.71 (0.743) |  | 0.137 | 0.084 | 1.630 | 0.104 |
|  | Total | 618 | 1.67 (0.817) |  |  |  |  |  |
| *One-way ANOVA  **regression model adjusted for years in current employment and since registration, completed any breastfeeding education, recommend breastfeeding to mothers, breastfed own children or intend to do so in the future  p significant <0.05  all 5-point Likert scale items; higher mean score indicates higher concrete knowledge | | | | | | | | |
