# Supplementary material for: Breastfeeding related knowledge, attitudes, perceptions and practices of primary healthcare professionals in Ireland: A national cross-sectional survey
Source: PLoS One. 2025 Apr 9;20(4):e0320763. doi: 10.1371/journal.pone.0320763 (PMC11981121; doi:10.1371/journal.pone.0320763)
Supplement: S3 Table — (DOCX) [file pone.0320763.s004.docx]

**S 3 Table Perceived attitudes and beliefs about breastfeeding among GPs, GP trainees and GP nurses**

| **Item/Variable** | **Professional role** | **N** | **One-way Anova** | | **Regression analysis**** | | | |
| --- | --- | --- | --- | --- | --- | --- | --- | --- |
|  |  |  | **Mean (SD)** | **p value** | **Unstandardized β Coefficients** | **Std. Error** | **t** | **p value** |
| **a) I am in favour of exclusive breastfeeding. (exclusive breastfeeding means no formula milk provided)** | GP | 352 | 4.17 (0.9) | <0.01* | 2.822 | 0.360 | 7.842 | 0.000 |
|  | GP Trainee | 88 | 4.24 (0.922) |  | -0.041 | 0.121 | -0.335 | 0.738 |
|  | GP Nurse | 166 | 3.73 (1.047) |  | -0.419 | 0.097 | -4.326 | 0.000 |
|  | Total | 606 | 4.06 (0.966) |  |  |  |  |  |
| **b) I am in favour of breastfeeding combined with formula milk**  (for this statement lower mean score indicates ideal attitude) | GP | 352 | 3.26 (0.916) | 0.018* | 4.222 | 0.351 | 12.044 | 0.000 |
|  | GP Trainee | 88 | 3.57 (0.907) |  | 0.127 | 0.118 | 1.073 | 0.284 |
|  | GP Nurse | 166 | 3.28 (0.907) |  | 0.104 | 0.094 | 1.100 | 0.272 |
|  | Total | 606 | 3.31 (0.917) |  |  |  |  |  |
| **c) I am in favour of breastfeeding in public** | GP | 352 | 4.69 (0.598) | 0.603* | 3.965 | 0.223 | 17.805 | 0.000 |
|  | GP Trainee | 88 | 4.73 (0.519) |  | -0.070 | 0.075 | -0.929 | 0.353 |
|  | GP Nurse | 166 | 4.65 (0.612) |  | 0.040 | 0.060 | 0.669 | 0.504 |
|  | Total | 606 | 4.68 (0.591) |  |  |  |  |  |
| **d) I am in favour of breastfeeding while returning to work** | GP | 352 | 4.47 (0.695) | 0.127* | 3.907 | 0.267 | 14.649 | 0.000 |
|  | GP Trainee | 88 | 4.63 (0.553) |  | 0.109 | 0.090 | 1.215 | 0.225 |
|  | GP Nurse | 166 | 4.45 (0.735) |  | 0.051 | 0.072 | 0.713 | 0.476 |
|  | Total | 606 | 4.49 (0.689) |  |  |  |  |  |
| **e) Breastfeeding has an impact on the social life of a mother**  (for this statement lower mean score indicates ideal attitude) | GP | 352 | 3.53 (1.117) | <0.01* | 4.916 | 0.433 | 11.358 | 0.000 |
|  | GP Trainee | 88 | 3.98 (1.05) |  | 0.146 | 0.146 | 1.001 | 0.317 |
|  | GP Nurse | 166 | 3.21 (1.2) |  | -0.272 | 0.117 | -2.332 | 0.020 |
|  | Total | 606 | 3.5 (1.154) |  |  |  |  |  |
| **f) Breastfeeding has an impact on the professional life of a mother**  (for this statement lower mean score indicates ideal attitude) | GP | 352 | 3.6 (1.041) | <0.01* | 4.722 | 0.422 | 11.183 | 0.000 |
|  | GP Trainee | 88 | 3.92 (1.074) |  | 0.103 | 0.142 | 0.721 | 0.471 |
|  | GP Nurse | 166 | 3.17 (1.211) |  | -0.315 | 0.114 | -2.765 | 0.006 |
|  | Total | 606 | 3.53 (1.12) |  |  |  |  |  |
| **g) Breastfeeding makes the father/partner feel isolated from raising their child**  (for this statement lower mean score indicates ideal attitude) | GP | 352 | 2.05 (0.928) | 0.510* | 2.884 | 0.364 | 7.922 | 0.000 |
|  | GP Trainee | 88 | 1.94 (0.914) |  | -0.115 | 0.123 | -0.935 | 0.350 |
|  | GP Nurse | 166 | 2.08 (0.987) |  | 0.026 | 0.098 | 0.266 | 0.790 |
|  | Total | 606 | 2.05 (0.942) |  |  |  |  |  |
| **h) A daily formula milk top-up has an impact on exclusive breastfeeding** | GP | 352 | 3.52 (1.078) | 0.177* | 2.670 | 0.410 | 6.515 | 0.000 |
|  | GP Trainee | 88 | 3.57 (1.037) |  | 0.009 | 0.138 | 0.066 | 0.947 |
|  | GP Nurse | 166 | 3.36 (1.027) |  | -0.228 | 0.110 | -2.067 | 0.039 |
|  | Total | 606 | 3.48 (1.06) |  |  |  |  |  |
| **i) Breastfeeding is more convenient and cheaper than formula milk** | GP | 352 | 4.44 (0.764) | <0.01* | 3.170 | 0.317 | 10.011 | 0.000 |
|  | GP Trainee | 88 | 3.99 (1.16) |  | -0.322 | 0.107 | -3.018 | 0.003 |
|  | GP Nurse | 166 | 4.43 (0.742) |  | -0.055 | 0.085 | -0.644 | 0.520 |
|  | Total | 606 | 4.37 (0.841) |  |  |  |  |  |
| **j) Mothers with excess milk should be encouraged to donate their milk to maternal/donor milk banks** | GP | 352 | 3.67 (0.724) | 0.427* | 3.351 | 0.293 | 11.449 | 0.000 |
|  | GP Trainee | 88 | 3.56 (0.741) |  | -0.163 | 0.099 | -1.657 | 0.098 |
|  | GP Nurse | 166 | 3.67 (0.812) |  | 0.047 | 0.079 | 0.596 | 0.552 |
|  | Total | 606 | 3.65 (0.751) |  |  |  |  |  |
| **k) I have the time to inform antenatal pregnant women about the importance of breastfeeding/risks of not breastfeeding** | GP | 352 | 3.13 (1.117) | 0.397* | 2.145 | 0.429 | 4.995 | 0.000 |
|  | GP Trainee | 88 | 3.03 (1.129) |  | 0.117 | 0.145 | 0.808 | 0.419 |
|  | GP Nurse | 166 | 2.99 (1.12) |  | -0.246 | 0.116 | -2.129 | 0.034 |
|  | Total | 606 | 3.08 (1.119) |  |  |  |  |  |
| **l) Low breastfeeding rates in Ireland are due to healthcare professionals not informing mothers about breastfeeding**  (for this statement lower mean score indicates ideal attitude) | GP | 352 | 2.59 (1.012) | <0.01* | 2.442 | 0.400 | 6.104 | 0.000 |
|  | GP Trainee | 88 | 2.64 (1.03) |  | -0.054 | 0.135 | -0.402 | 0.688 |
|  | GP Nurse | 166 | 2.91 (1.049) |  | 0.378 | 0.108 | 3.504 | 0.000 |
|  | Total | 606 | 2.68 (1.033) |  |  |  |  |  |
| *One-way ANOVA  **regression model adjusted for years in current employment and since registration, completed any breastfeeding education, recommend breastfeeding to mothers, breastfed own children or intend to do so in the future  p significant <0.05  all 5-point Likert scale items; higher mean score indicates stronger agreement to the statement | | | | | | | | |
