# Supplementary material for: Breastfeeding related knowledge, attitudes, perceptions and practices of primary healthcare professionals in Ireland: A national cross-sectional survey
Source: PLoS One. 2025 Apr 9;20(4):e0320763. doi: 10.1371/journal.pone.0320763 (PMC11981121; doi:10.1371/journal.pone.0320763)
Supplement: S4 Table — (DOCX) [file pone.0320763.s005.docx]

**S4 Table Perceived confidence with breastfeeding related issues among GPs, GP trainees and GP nurses**

| **Item/Variable** | **Professional role** |  | **One-way Anova** | | **Regression analysis**** | | | |
| --- | --- | --- | --- | --- | --- | --- | --- | --- |
|  |  | **N** | **Mean (SD)** | **p value** | **Unstandardized β Coefficients** | **Std. Error** | **t** | **p value** |
| **a) Latching problems** | GP | 349 | 2.12 (0.699) | <0.01 | 0.934 | 0.268 | 3.483 | 0.001 |
|  | GP Trainee | 88 | 1.88 (0.724) |  | -0.043 | 0.090 | -0.473 | 0.636 |
|  | GP Nurse | 165 | 1.98 (0.773) |  | -0.219 | 0.072 | -3.034 | 0.003 |
|  | Total | 602 | 2.04 (0.728) |  |  |  |  |  |
| **b) Supporting mothers with breast engorgement or full breasts** | GP | 349 | 2.41 (0.675) | <0.01 | 1.510 | 0.273 | 5.528 | 0.000 |
|  | GP Trainee | 88 | 2.14 (0.79) |  | -0.079 | 0.092 | -0.853 | 0.394 |
|  | GP Nurse | 165 | 2.07 (0.805) |  | -0.466 | 0.074 | -6.324 | 0.000 |
|  | Total | 602 | 2.28 (0.746) |  |  |  |  |  |
| **c) Recognising and managing nipple problems such as mastitis and nipple thrush** | GP | 349 | 2.73 (0.483) | <0.01 | 2.183 | 0.230 | 9.487 | 0.000 |
|  | GP Trainee | 88 | 2.41 (0.721) |  | -0.263 | 0.078 | -3.393 | 0.001 |
|  | GP Nurse | 165 | 2.19 (0.78) |  | -0.601 | 0.062 | -9.681 | 0.000 |
|  | Total | 602 | 2.53 (0.657) |  |  |  |  |  |
| **d) Reflux and the breastfed baby** | GP | 349 | 2.43 (0.581) | <0.01 | 2.232 | 0.250 | 8.925 | 0.000 |
|  | GP Trainee | 88 | 1.99 (0.634) |  | -0.334 | 0.084 | -3.964 | 0.000 |
|  | GP Nurse | 165 | 1.81 (0.793) |  | -0.699 | 0.067 | -10.37 | 0.000 |
|  | Total | 602 | 2.19 (0.709) |  |  |  |  |  |
| **e) Supporting lactation following preterm birth (Preterm means prior to 37 weeks)** | GP | 349 | 1.83 (0.719) | <0.01 | 1.191 | 0.271 | 4.387 | 0.000 |
|  | GP Trainee | 88 | 1.49 (0.678) |  | -0.194 | 0.092 | -2.122 | 0.034 |
|  | GP Nurse | 165 | 1.66 (0.753) |  | -0.279 | 0.073 | -3.809 | 0.000 |
|  | Total | 602 | 1.73 (0.732) |  |  |  |  |  |
| **f) Supporting lactation suppression, e.g., following infant loss or maternal decision to stop breastfeeding.** | GP | 349 | 1.83 (0.724) | <0.01 | 1.800 | 0.271 | 6.646 | 0.000 |
|  | GP Trainee | 88 | 1.36 (0.571) |  | -0.270 | 0.091 | -2.955 | 0.003 |
|  | GP Nurse | 165 | 1.59 (0.781) |  | -0.356 | 0.073 | -4.880 | 0.000 |
|  | Total | 602 | 1.7 (0.74) |  |  |  |  |  |
| *One-way ANOVA  **regression model adjusted for years in current employment and since registration, completed any breastfeeding education, recommend breastfeeding to mothers, breastfed own children or intend to do so in the future  p significant <0.05  all 3-point Likert scale items; higher mean score indicates higher perceived confidence | | | | | | | | |
