# Supplementary material for: Breastfeeding related knowledge, attitudes, perceptions and practices of primary healthcare professionals in Ireland: A national cross-sectional survey
Source: PLoS One. 2025 Apr 9;20(4):e0320763. doi: 10.1371/journal.pone.0320763 (PMC11981121; doi:10.1371/journal.pone.0320763)
Supplement: Table S5-S10 — (DOCX) [file pone.0320763.s006.docx]

**Table S5: Testing normal distribution for Likert scale items – respondents’ perceived and factual knowledge of breastfeeding**

| **Descriptives** | | | | |
| --- | --- | --- | --- | --- |
|  | | | Statistic | Std. Error |
| a) I am confident with my knowledge about breastfeeding | Mean | | 3.56 | .044 |
|  | 95% Confidence Interval for Mean | Lower Bound | 3.47 |  |
|  |  | Upper Bound | 3.65 |  |
|  | 5% Trimmed Mean | | 3.62 |  |
|  | Median | | 4.00 |  |
|  | Variance | | 1.223 |  |
|  | Std. Deviation | | 1.106 |  |
|  | Minimum | | 1 |  |
|  | Maximum | | 5 |  |
|  | Range | | 4 |  |
|  | Interquartile Range | | 1 |  |
|  | Skewness | | -.805 | .098 |
|  | Kurtosis | | -.142 | .196 |
| b) I have obtained most of my knowledge about breastfeeding through my own personal research | Mean | | 3.98 | .039 |
|  | 95% Confidence Interval for Mean | Lower Bound | 3.91 |  |
|  |  | Upper Bound | 4.06 |  |
|  | 5% Trimmed Mean | | 4.07 |  |
|  | Median | | 4.00 |  |
|  | Variance | | .928 |  |
|  | Std. Deviation | | .964 |  |
|  | Minimum | | 1 |  |
|  | Maximum | | 5 |  |
|  | Range | | 4 |  |
|  | Interquartile Range | | 1 |  |
|  | Skewness | | -1.219 | .098 |
|  | Kurtosis | | 1.421 | .196 |
| c) My level of knowledge could be improved | Mean | | 4.16 | .033 |
|  | 95% Confidence Interval for Mean | Lower Bound | 4.09 |  |
|  |  | Upper Bound | 4.22 |  |
|  | 5% Trimmed Mean | | 4.25 |  |
|  | Median | | 4.00 |  |
|  | Variance | | .671 |  |
|  | Std. Deviation | | .819 |  |
|  | Minimum | | 1 |  |
|  | Maximum | | 5 |  |
|  | Range | | 4 |  |
|  | Interquartile Range | | 1 |  |
|  | Skewness | | -1.416 | .098 |
|  | Kurtosis | | 3.127 | .196 |
| d) I am confident that I can manage breastfeeding related issues in my everyday practice | Mean | | 3.45 | .042 |
|  | 95% Confidence Interval for Mean | Lower Bound | 3.37 |  |
|  |  | Upper Bound | 3.54 |  |
|  | 5% Trimmed Mean | | 3.50 |  |
|  | Median | | 4.00 |  |
|  | Variance | | 1.111 |  |
|  | Std. Deviation | | 1.054 |  |
|  | Minimum | | 1 |  |
|  | Maximum | | 5 |  |
|  | Range | | 4 |  |
|  | Interquartile Range | | 1 |  |
|  | Skewness | | -.580 | .098 |
|  | Kurtosis | | -.419 | .196 |
| e) Formula milk is easier to digest than maternal milk | Mean | | 1.54 | .029 |
|  | 95% Confidence Interval for Mean | Lower Bound | 1.48 |  |
|  |  | Upper Bound | 1.60 |  |
|  | 5% Trimmed Mean | | 1.47 |  |
|  | Median | | 1.00 |  |
|  | Variance | | .505 |  |
|  | Std. Deviation | | .711 |  |
|  | Minimum | | 1 |  |
|  | Maximum | | 5 |  |
|  | Range | | 4 |  |
|  | Interquartile Range | | 1 |  |
|  | Skewness | | 1.502 | .098 |
|  | Kurtosis | | 3.259 | .196 |
| f) A breastfeeding mother should avoid alcohol | Mean | | 3.27 | .047 |
|  | 95% Confidence Interval for Mean | Lower Bound | 3.18 |  |
|  |  | Upper Bound | 3.37 |  |
|  | 5% Trimmed Mean | | 3.30 |  |
|  | Median | | 4.00 |  |
|  | Variance | | 1.379 |  |
|  | Std. Deviation | | 1.174 |  |
|  | Minimum | | 1 |  |
|  | Maximum | | 5 |  |
|  | Range | | 4 |  |
|  | Interquartile Range | | 2 |  |
|  | Skewness | | -.243 | .098 |
|  | Kurtosis | | -1.045 | .196 |
| g) A carrier of Hepatitis B who has been vaccinated can safely breastfeed | Mean | | 3.69 | .032 |
|  | 95% Confidence Interval for Mean | Lower Bound | 3.63 |  |
|  |  | Upper Bound | 3.76 |  |
|  | 5% Trimmed Mean | | 3.73 |  |
|  | Median | | 4.00 |  |
|  | Variance | | .641 |  |
|  | Std. Deviation | | .800 |  |
|  | Minimum | | 1 |  |
|  | Maximum | | 5 |  |
|  | Range | | 4 |  |
|  | Interquartile Range | | 1 |  |
|  | Skewness | | -.783 | .098 |
|  | Kurtosis | | .847 | .196 |
| h) A carrier of HIV can transfer the virus to her baby through breastfeeding | Mean | | 3.22 | .042 |
|  | 95% Confidence Interval for Mean | Lower Bound | 3.14 |  |
|  |  | Upper Bound | 3.30 |  |
|  | 5% Trimmed Mean | | 3.24 |  |
|  | Median | | 3.00 |  |
|  | Variance | | 1.093 |  |
|  | Std. Deviation | | 1.046 |  |
|  | Minimum | | 1 |  |
|  | Maximum | | 5 |  |
|  | Range | | 4 |  |
|  | Interquartile Range | | 2 |  |
|  | Skewness | | -.231 | .098 |
|  | Kurtosis | | -.747 | .196 |
| i)A mother with a fever &gt; 38C should temporarily interrupt breastfeeding | Mean | | 1.90 | .035 |
|  | 95% Confidence Interval for Mean | Lower Bound | 1.83 |  |
|  |  | Upper Bound | 1.97 |  |
|  | 5% Trimmed Mean | | 1.83 |  |
|  | Median | | 2.00 |  |
|  | Variance | | .778 |  |
|  | Std. Deviation | | .882 |  |
|  | Minimum | | 1 |  |
|  | Maximum | | 5 |  |
|  | Range | | 4 |  |
|  | Interquartile Range | | 1 |  |
|  | Skewness | | 1.057 | .098 |
|  | Kurtosis | | .931 | .196 |
| j) A mother with mastitis should stop breastfeeding | Mean | | 1.56 | .032 |
|  | 95% Confidence Interval for Mean | Lower Bound | 1.50 |  |
|  |  | Upper Bound | 1.63 |  |
|  | 5% Trimmed Mean | | 1.46 |  |
|  | Median | | 1.00 |  |
|  | Variance | | .616 |  |
|  | Std. Deviation | | .785 |  |
|  | Minimum | | 1 |  |
|  | Maximum | | 5 |  |
|  | Range | | 4 |  |
|  | Interquartile Range | | 1 |  |
|  | Skewness | | 1.808 | .098 |
|  | Kurtosis | | 3.956 | .196 |
| k) Breastfeeding should continue if the mother smokes | Mean | | 3.85 | .038 |
|  | 95% Confidence Interval for Mean | Lower Bound | 3.78 |  |
|  |  | Upper Bound | 3.93 |  |
|  | 5% Trimmed Mean | | 3.92 |  |
|  | Median | | 4.00 |  |
|  | Variance | | .880 |  |
|  | Std. Deviation | | .938 |  |
|  | Minimum | | 1 |  |
|  | Maximum | | 5 |  |
|  | Range | | 4 |  |
|  | Interquartile Range | | 0 |  |
|  | Skewness | | -1.030 | .098 |
|  | Kurtosis | | .921 | .196 |
| l) I am confident discussing safe medication use with breastfeeding mothers | Mean | | 3.46 | .042 |
|  | 95% Confidence Interval for Mean | Lower Bound | 3.38 |  |
|  |  | Upper Bound | 3.55 |  |
|  | 5% Trimmed Mean | | 3.52 |  |
|  | Median | | 4.00 |  |
|  | Variance | | 1.066 |  |
|  | Std. Deviation | | 1.032 |  |
|  | Minimum | | 1 |  |
|  | Maximum | | 5 |  |
|  | Range | | 4 |  |
|  | Interquartile Range | | 1 |  |
|  | Skewness | | -.720 | .098 |
|  | Kurtosis | | -.180 | .196 |
| m) Breast surgeries i.e., augmentation or reduction make breastfeeding difficult | Mean | | 3.17 | .034 |
|  | 95% Confidence Interval for Mean | Lower Bound | 3.11 |  |
|  |  | Upper Bound | 3.24 |  |
|  | 5% Trimmed Mean | | 3.18 |  |
|  | Median | | 3.00 |  |
|  | Variance | | .706 |  |
|  | Std. Deviation | | .840 |  |
|  | Minimum | | 1 |  |
|  | Maximum | | 5 |  |
|  | Range | | 4 |  |
|  | Interquartile Range | | 1 |  |
|  | Skewness | | -.267 | .098 |
|  | Kurtosis | | -.458 | .196 |
| n) Breastfed babies are less likely to suffer reflux | Mean | | 3.57 | .038 |
|  | 95% Confidence Interval for Mean | Lower Bound | 3.50 |  |
|  |  | Upper Bound | 3.64 |  |
|  | 5% Trimmed Mean | | 3.60 |  |
|  | Median | | 4.00 |  |
|  | Variance | | .887 |  |
|  | Std. Deviation | | .942 |  |
|  | Minimum | | 1 |  |
|  | Maximum | | 5 |  |
|  | Range | | 4 |  |
|  | Interquartile Range | | 1 |  |
|  | Skewness | | -.599 | .098 |
|  | Kurtosis | | -.033 | .196 |
| o) Breastfeeding mothers need to night wean at 6 months | Mean | | 1.67 | .033 |
|  | 95% Confidence Interval for Mean | Lower Bound | 1.60 |  |
|  |  | Upper Bound | 1.73 |  |
|  | 5% Trimmed Mean | | 1.58 |  |
|  | Median | | 1.00 |  |
|  | Variance | | .667 |  |
|  | Std. Deviation | | .817 |  |
|  | Minimum | | 1 |  |
|  | Maximum | | 5 |  |
|  | Range | | 4 |  |
|  | Interquartile Range | | 1 |  |
|  | Skewness | | 1.259 | .098 |
|  | Kurtosis | | 1.549 | .196 |

**Table S6: Testing normal distribution for Likert scale items – respondents’ perceived and factual knowledge of breastfeeding**

| **Tests of Normality** | | | | | | |
| --- | --- | --- | --- | --- | --- | --- |
|  | Kolmogorov-Smirnov^a^ | | | Shapiro-Wilk | | |
|  | Statistic | df | Sig. | Statistic | df | Sig. |
| a) I am confident with my knowledge about breastfeeding | .318 | 618 | .000 | .840 | 618 | .000 |
| b) I have obtained most of my knowledge about breastfeeding through my own personal research | .320 | 618 | .000 | .790 | 618 | .000 |
| c) My level of knowledge could be improved | .306 | 618 | .000 | .751 | 618 | .000 |
| d) I am confident that I can manage breastfeeding related issues in my everyday practice | .288 | 618 | .000 | .869 | 618 | .000 |
| e) Formula milk is easier to digest than maternal milk | .336 | 618 | .000 | .708 | 618 | .000 |
| f) A breastfeeding mother should avoid alcohol | .248 | 618 | .000 | .886 | 618 | .000 |
| g) A carrier of Hepatitis B who has been vaccinated can safely breastfeed | .332 | 618 | .000 | .821 | 618 | .000 |
| h) A carrier of HIV can transfer the virus to her baby through breastfeeding | .228 | 618 | .000 | .898 | 618 | .000 |
| i)A mother with a fever &gt; 38C should temporarily interrupt breastfeeding | .290 | 618 | .000 | .795 | 618 | .000 |
| j) A mother with mastitis should stop breastfeeding | .323 | 618 | .000 | .678 | 618 | .000 |
| k) Breastfeeding should continue if the mother smokes | .334 | 618 | .000 | .809 | 618 | .000 |
| l) I am confident discussing safe medication use with breastfeeding mothers | .310 | 618 | .000 | .849 | 618 | .000 |
| m) Breast surgeries i.e., augmentation or reduction make breastfeeding difficult | .220 | 618 | .000 | .868 | 618 | .000 |
| n) Breastfed babies are less likely to suffer reflux | .288 | 618 | .000 | .867 | 618 | .000 |
| o) Breastfeeding mothers need to night wean at 6 months | .304 | 618 | .000 | .755 | 618 | .000 |
| a. Lilliefors Significance Correction | | | | | | |

**Table S7: Testing normal distribution for Likert scale items – respondents’ attitude towards breastfeeding**

| **Descriptives** | | | | |
| --- | --- | --- | --- | --- |
|  | | | Statistic | Std. Error |
| a) I am in favour of exclusive breastfeeding? (exclusive breastfeeding means no formula milk provided) | Mean | | 4.06 | .039 |
|  | 95% Confidence Interval for Mean | Lower Bound | 3.98 |  |
|  |  | Upper Bound | 4.14 |  |
|  | 5% Trimmed Mean | | 4.14 |  |
|  | Median | | 4.00 |  |
|  | Variance | | .933 |  |
|  | Std. Deviation | | .966 |  |
|  | Minimum | | 1 |  |
|  | Maximum | | 5 |  |
|  | Range | | 4 |  |
|  | Interquartile Range | | 1 |  |
|  | Skewness | | -.906 | .099 |
|  | Kurtosis | | .232 | .198 |
| b) I am in favour of breastfeeding combined with formula milk | Mean | | 3.31 | .037 |
|  | 95% Confidence Interval for Mean | Lower Bound | 3.24 |  |
|  |  | Upper Bound | 3.39 |  |
|  | 5% Trimmed Mean | | 3.33 |  |
|  | Median | | 3.00 |  |
|  | Variance | | .840 |  |
|  | Std. Deviation | | .917 |  |
|  | Minimum | | 1 |  |
|  | Maximum | | 5 |  |
|  | Range | | 4 |  |
|  | Interquartile Range | | 1 |  |
|  | Skewness | | -.415 | .099 |
|  | Kurtosis | | -.150 | .198 |
| c) I am in favour of breastfeeding in public | Mean | | 4.68 | .024 |
|  | 95% Confidence Interval for Mean | Lower Bound | 4.64 |  |
|  |  | Upper Bound | 4.73 |  |
|  | 5% Trimmed Mean | | 4.76 |  |
|  | Median | | 5.00 |  |
|  | Variance | | .349 |  |
|  | Std. Deviation | | .591 |  |
|  | Minimum | | 1 |  |
|  | Maximum | | 5 |  |
|  | Range | | 4 |  |
|  | Interquartile Range | | 1 |  |
|  | Skewness | | -2.381 | .099 |
|  | Kurtosis | | 8.215 | .198 |
| d) I am in favour of breastfeeding while returning to work | Mean | | 4.49 | .028 |
|  | 95% Confidence Interval for Mean | Lower Bound | 4.43 |  |
|  |  | Upper Bound | 4.54 |  |
|  | 5% Trimmed Mean | | 4.56 |  |
|  | Median | | 5.00 |  |
|  | Variance | | .475 |  |
|  | Std. Deviation | | .689 |  |
|  | Minimum | | 1 |  |
|  | Maximum | | 5 |  |
|  | Range | | 4 |  |
|  | Interquartile Range | | 1 |  |
|  | Skewness | | -1.417 | .099 |
|  | Kurtosis | | 2.523 | .198 |
| e) Breastfeeding has an impact on the social life of a mother | Mean | | 3.50 | .047 |
|  | 95% Confidence Interval for Mean | Lower Bound | 3.41 |  |
|  |  | Upper Bound | 3.60 |  |
|  | 5% Trimmed Mean | | 3.56 |  |
|  | Median | | 4.00 |  |
|  | Variance | | 1.331 |  |
|  | Std. Deviation | | 1.154 |  |
|  | Minimum | | 1 |  |
|  | Maximum | | 5 |  |
|  | Range | | 4 |  |
|  | Interquartile Range | | 1 |  |
|  | Skewness | | -.527 | .099 |
|  | Kurtosis | | -.662 | .198 |
| f) Breastfeeding has an impact on the professional life of a mother | Mean | | 3.53 | .045 |
|  | 95% Confidence Interval for Mean | Lower Bound | 3.44 |  |
|  |  | Upper Bound | 3.62 |  |
|  | 5% Trimmed Mean | | 3.59 |  |
|  | Median | | 4.00 |  |
|  | Variance | | 1.254 |  |
|  | Std. Deviation | | 1.120 |  |
|  | Minimum | | 1 |  |
|  | Maximum | | 5 |  |
|  | Range | | 4 |  |
|  | Interquartile Range | | 1 |  |
|  | Skewness | | -.659 | .099 |
|  | Kurtosis | | -.401 | .198 |
| g) Breastfeeding makes the father/partner feel isolated from raising their child | Mean | | 2.05 | .038 |
|  | 95% Confidence Interval for Mean | Lower Bound | 1.97 |  |
|  |  | Upper Bound | 2.12 |  |
|  | 5% Trimmed Mean | | 1.99 |  |
|  | Median | | 2.00 |  |
|  | Variance | | .887 |  |
|  | Std. Deviation | | .942 |  |
|  | Minimum | | 1 |  |
|  | Maximum | | 5 |  |
|  | Range | | 4 |  |
|  | Interquartile Range | | 2 |  |
|  | Skewness | | .670 | .099 |
|  | Kurtosis | | -.239 | .198 |
| h) A daily formula milk top-up has an impact on exclusive breastfeeding | Mean | | 3.48 | .043 |
|  | 95% Confidence Interval for Mean | Lower Bound | 3.40 |  |
|  |  | Upper Bound | 3.57 |  |
|  | 5% Trimmed Mean | | 3.52 |  |
|  | Median | | 4.00 |  |
|  | Variance | | 1.123 |  |
|  | Std. Deviation | | 1.060 |  |
|  | Minimum | | 1 |  |
|  | Maximum | | 5 |  |
|  | Range | | 4 |  |
|  | Interquartile Range | | 1 |  |
|  | Skewness | | -.475 | .099 |
|  | Kurtosis | | -.517 | .198 |
| i) Breastfeeding is more convenient and cheaper than formula milk | Mean | | 4.37 | .034 |
|  | 95% Confidence Interval for Mean | Lower Bound | 4.31 |  |
|  |  | Upper Bound | 4.44 |  |
|  | 5% Trimmed Mean | | 4.48 |  |
|  | Median | | 5.00 |  |
|  | Variance | | .707 |  |
|  | Std. Deviation | | .841 |  |
|  | Minimum | | 1 |  |
|  | Maximum | | 5 |  |
|  | Range | | 4 |  |
|  | Interquartile Range | | 1 |  |
|  | Skewness | | -1.629 | .099 |
|  | Kurtosis | | 3.019 | .198 |
| j) Mothers with excess milk should be encouraged to donate their milk to maternal/donor milk banks | Mean | | 3.65 | .031 |
|  | 95% Confidence Interval for Mean | Lower Bound | 3.59 |  |
|  |  | Upper Bound | 3.71 |  |
|  | 5% Trimmed Mean | | 3.66 |  |
|  | Median | | 4.00 |  |
|  | Variance | | .564 |  |
|  | Std. Deviation | | .751 |  |
|  | Minimum | | 1 |  |
|  | Maximum | | 5 |  |
|  | Range | | 4 |  |
|  | Interquartile Range | | 1 |  |
|  | Skewness | | -.063 | .099 |
|  | Kurtosis | | .035 | .198 |
| k) I have the time to inform antenatal pregnant women about the importance of breastfeeding/risks of not breastfeeding | Mean | | 3.08 | .045 |
|  | 95% Confidence Interval for Mean | Lower Bound | 2.99 |  |
|  |  | Upper Bound | 3.17 |  |
|  | 5% Trimmed Mean | | 3.09 |  |
|  | Median | | 3.00 |  |
|  | Variance | | 1.253 |  |
|  | Std. Deviation | | 1.119 |  |
|  | Minimum | | 1 |  |
|  | Maximum | | 5 |  |
|  | Range | | 4 |  |
|  | Interquartile Range | | 2 |  |
|  | Skewness | | -.135 | .099 |
|  | Kurtosis | | -1.036 | .198 |
| l) Low breastfeeding rates in Ireland are due to healthcare professionals not informing mothers about breastfeeding | Mean | | 2.68 | .042 |
|  | 95% Confidence Interval for Mean | Lower Bound | 2.60 |  |
|  |  | Upper Bound | 2.77 |  |
|  | 5% Trimmed Mean | | 2.66 |  |
|  | Median | | 3.00 |  |
|  | Variance | | 1.066 |  |
|  | Std. Deviation | | 1.033 |  |
|  | Minimum | | 1 |  |
|  | Maximum | | 5 |  |
|  | Range | | 4 |  |
|  | Interquartile Range | | 1 |  |
|  | Skewness | | .221 | .099 |
|  | Kurtosis | | -.612 | .198 |

**Table S8: Testing normal distribution for Likert scale items – respondents’ attitude towards breastfeeding**

| **Tests of Normality** | | | | | | |
| --- | --- | --- | --- | --- | --- | --- |
|  | Kolmogorov-Smirnov^a^ | | | Shapiro-Wilk | | |
|  | Statistic | df | Sig. | Statistic | df | Sig. |
| a) I am in favour of exclusive breastfeeding? (exclusive breastfeeding means no formula milk provided) | .237 | 606 | .000 | .823 | 606 | .000 |
| b) I am in favour of breastfeeding combined with formula milk | .237 | 606 | .000 | .882 | 606 | .000 |
| c) I am in favour of breastfeeding in public | .435 | 606 | .000 | .565 | 606 | .000 |
| d) I am in favour of breastfeeding while returning to work | .352 | 606 | .000 | .703 | 606 | .000 |
| e) Breastfeeding has an impact on the social life of a mother | .265 | 606 | .000 | .878 | 606 | .000 |
| f) Breastfeeding has an impact on the professional life of a mother | .291 | 606 | .000 | .864 | 606 | .000 |
| g) Breastfeeding makes the father/partner feel isolated from raising their child | .249 | 606 | .000 | .848 | 606 | .000 |
| h) A daily formula milk top-up has an impact on exclusive breastfeeding | .261 | 606 | .000 | .886 | 606 | .000 |
| i) Breastfeeding is more convenient and cheaper than formula milk | .312 | 606 | .000 | .716 | 606 | .000 |
| j) Mothers with excess milk should be encouraged to donate their milk to maternal/donor milk banks | .250 | 606 | .000 | .843 | 606 | .000 |
| k) I have the time to inform antenatal pregnant women about the importance of breastfeeding/risks of not breastfeeding | .235 | 606 | .000 | .888 | 606 | .000 |
| l) Low breastfeeding rates in Ireland are due to healthcare professionals not informing mothers about breastfeeding | .211 | 606 | .000 | .905 | 606 | .000 |
| a. Lilliefors Significance Correction | | | | | | |

**Table S9: Testing normal distribution for Likert scale items – respondents’ confidence with breastfeeding related issues**

| **Descriptives** | | | | |
| --- | --- | --- | --- | --- |
|  | | | Statistic | Std. Error |
| a) Latching problems | Mean | | 1.96 | .030 |
|  | 95% Confidence Interval for Mean | Lower Bound | 1.90 |  |
|  |  | Upper Bound | 2.02 |  |
|  | 5% Trimmed Mean | | 1.95 |  |
|  | Median | | 2.00 |  |
|  | Variance | | .531 |  |
|  | Std. Deviation | | .728 |  |
|  | Minimum | | 1 |  |
|  | Maximum | | 3 |  |
|  | Range | | 2 |  |
|  | Interquartile Range | | 1 |  |
|  | Skewness | | .066 | .100 |
|  | Kurtosis | | -1.110 | .199 |
| b) Supporting mothers with breast engorgement or full breasts | Mean | | 1.72 | .030 |
|  | 95% Confidence Interval for Mean | Lower Bound | 1.66 |  |
|  |  | Upper Bound | 1.78 |  |
|  | 5% Trimmed Mean | | 1.69 |  |
|  | Median | | 2.00 |  |
|  | Variance | | .556 |  |
|  | Std. Deviation | | .746 |  |
|  | Minimum | | 1 |  |
|  | Maximum | | 3 |  |
|  | Range | | 2 |  |
|  | Interquartile Range | | 1 |  |
|  | Skewness | | .496 | .100 |
|  | Kurtosis | | -1.060 | .199 |
| c) Recognising and managing nipple problems such as mastitis and nipple thrush | Mean | | 1.47 | .027 |
|  | 95% Confidence Interval for Mean | Lower Bound | 1.41 |  |
|  |  | Upper Bound | 1.52 |  |
|  | 5% Trimmed Mean | | 1.41 |  |
|  | Median | | 1.00 |  |
|  | Variance | | .432 |  |
|  | Std. Deviation | | .657 |  |
|  | Minimum | | 1 |  |
|  | Maximum | | 3 |  |
|  | Range | | 2 |  |
|  | Interquartile Range | | 1 |  |
|  | Skewness | | 1.098 | .100 |
|  | Kurtosis | | .015 | .199 |
| d) Reflux and the breastfed baby | Mean | | 1.81 | .029 |
|  | 95% Confidence Interval for Mean | Lower Bound | 1.75 |  |
|  |  | Upper Bound | 1.86 |  |
|  | 5% Trimmed Mean | | 1.78 |  |
|  | Median | | 2.00 |  |
|  | Variance | | .503 |  |
|  | Std. Deviation | | .709 |  |
|  | Minimum | | 1 |  |
|  | Maximum | | 3 |  |
|  | Range | | 2 |  |
|  | Interquartile Range | | 1 |  |
|  | Skewness | | .298 | .100 |
|  | Kurtosis | | -.988 | .199 |
| e) Supporting lactation following preterm birth (Preterm means prior to 37 weeks) | Mean | | 2.27 | .030 |
|  | 95% Confidence Interval for Mean | Lower Bound | 2.21 |  |
|  |  | Upper Bound | 2.33 |  |
|  | 5% Trimmed Mean | | 2.30 |  |
|  | Median | | 2.00 |  |
|  | Variance | | .536 |  |
|  | Std. Deviation | | .732 |  |
|  | Minimum | | 1 |  |
|  | Maximum | | 3 |  |
|  | Range | | 2 |  |
|  | Interquartile Range | | 1 |  |
|  | Skewness | | -.463 | .100 |
|  | Kurtosis | | -1.023 | .199 |
| f) Supporting lactation suppression, e.g., following infant loss or maternal decision to stop breastfeeding. | Mean | | 2.30 | .030 |
|  | 95% Confidence Interval for Mean | Lower Bound | 2.24 |  |
|  |  | Upper Bound | 2.36 |  |
|  | 5% Trimmed Mean | | 2.34 |  |
|  | Median | | 2.00 |  |
|  | Variance | | .547 |  |
|  | Std. Deviation | | .740 |  |
|  | Minimum | | 1 |  |
|  | Maximum | | 3 |  |
|  | Range | | 2 |  |
|  | Interquartile Range | | 1 |  |
|  | Skewness | | -.548 | .100 |
|  | Kurtosis | | -.999 | .199 |

**Table S10: Testing normal distribution for Likert scale items – respondents’ confidence with breastfeeding related issues**

| **Tests of Normality** | | | | | | |
| --- | --- | --- | --- | --- | --- | --- |
|  | Kolmogorov-Smirnov^a^ | | | Shapiro-Wilk | | |
|  | Statistic | df | Sig. | Statistic | df | Sig. |
| a) Latching problems | .236 | 602 | .000 | .809 | 602 | .000 |
| b) Supporting mothers with breast engorgement or full breasts | .288 | 602 | .000 | .776 | 602 | .000 |
| c) Recognising and managing nipple problems such as mastitis and nipple thrush | .387 | 602 | .000 | .683 | 602 | .000 |
| d) Reflux and the breastfed baby | .241 | 602 | .000 | .796 | 602 | .000 |
| e) Supporting lactation following preterm birth (Preterm means prior to 37 weeks) | .278 | 602 | .000 | .781 | 602 | .000 |
| f) Supporting lactation suppression, e.g., following infant loss or maternal decision to stop breastfeeding. | .297 | 602 | .000 | .770 | 602 | .000 |
| a. Lilliefors Significance Correction | | | | | | |
